# Variable disruption of epithelial monolayers by *Neisseria meningitidis* carriage isolates of the hypervirulent MenW cc11 and MenY cc23 lineages

**DOI:** 10.1099/mic.0.001305

**Published:** 2023-02-23

**Authors:** Neelam Dave, Raed S. Albiheyri, Joseph J. Wanford, Luke R. Green, Neil J. Oldfield, David P. J. Turner, Luisa Martinez-Pomares, Christopher D. Bayliss

**Affiliations:** ^1^​ Department of Genetics and Genome Biology, University of Leicester, Leicester, UK; ^2^​ School of Life Sciences, University of Nottingham, Nottingham, UK; ^†^​ Present address: Department of Biological Sciences, Faculty of Science, King Abdulaziz University, Jeddah, Saudi Arabia; ^‡^​ Present address: Department of Infectious Disease, King’s College, London, UK; ^§^​ Present address: Department of Infection, Immunity and Cardiovascular Disease, University of Sheffield, Sheffield, UK

**Keywords:** air–liquid interface, meningococcus, MenW, *Neisseria meningitidis*, opa, phase variation

## Abstract

Colonization of mucosal tissues by *Neisseria meningitidis* requires adhesion mediated by the type IV pilus and multiple outer-membrane proteins. Penetration of the mucosa and invasion of epithelial cells are thought to contribute to host persistence and invasive disease. Using Calu-3 cell monolayers grown at an air–liquid interface, we examined adhesion, invasion and monolayer disruption by carriage isolates of two clonal complexes of *N. meningitidis *. Carriage isolates of both the serogroup Y cc23 and the hypervirulent serogroup W cc11 lineages exhibited high levels of cellular adhesion, and a variable disruption phenotype across independent isolates. Inactivation of the gene encoding the main pilus sub-unit in multiple cc11 isolates abrogated both adhesive capacity and ability to disrupt epithelial monolayers. Contrastingly, inactivation of the phase-variable *opa* or *nadA* genes reduced adhesion and invasion, but not disruption of monolayer integrity. Adherence of tissue-disruptive meningococci correlated with loss of staining for the tight junction protein, occludin. Intriguingly, in a pilus-negative strain background, we observed compensatory ON switching of *opa* genes, which facilitated continued adhesion. We conclude that disruption of epithelial monolayers occurs in multiple meningococcal lineages but can vary during carriage and is intimately linked to pilus-mediated adhesion.

## Introduction

*Neisseria meningitidis* (the meningococcus, Nm) is a Gram-negative human pathogen that transiently colonizes the nasopharynx of 10–35 % of healthy individuals in the population [1, 2]. This ‘carriage’ state is a prerequisite for translocation of meningococci across the respiratory epithelium into the bloodstream and for systemic infections referred to as invasive meningococcal disease (IMD), including septicaemia and meningitis [3, 4]. Disease-causing Nm isolates are predominantly from six serogroups, A, B, C, W, X and Y, and from a restricted sub-set of clonal complexes [5–7]. The MenW ST-11 lineage is a major hypervirulent lineage that underwent a rapid global expansion between 2009 and 2016, whilst the MenY cc23 lineage is a globally distributed carriage-associated lineage that causes significant levels of disease, particularly among the elderly [6, 8].

A key stage of meningococcal carriage is attachment to, and formation of, microcolonies on mucosal surfaces [2, 9]. Epithelial adhesion by meningococci is predominantly mediated by the type IV pilus and outer-membrane proteins (OMPs). The type IV pilus mediates host cell attachment – with CD46 being implicated as a potential ligand on epithelial cells and CD147 on endothelial cells – and cell-to-cell interactions that contribute to microcolony formation [9, 10]. Energy-driven retraction of the pilus, mediated by PilT, contributes to these processes and also triggers cellular remodelling with formation of actin-philopodia from the membranes of colonized cells [8]. Meningococci encode two PilC proteins (PilC1 and PilC2) that are located on the outer membrane and regulate the retraction process, with both proteins having an inhibitory effect on retraction [11, 12]. These proteins may also contribute directly to adhesion [13]. The major Nm OMPs associated with adhesion are the opacity-associated proteins (Opa) that are encoded by four loci within Nm genomes [14]. These proteins exhibit allelic variation, with most variants mediating adhesion to human CEACAMs but with a sub-set binding to heparan sulphate proteoglycans (HSPGs) [1]. Opa variants have been shown to enable efficient colonization by meningococci of the upper respiratory tracts of transgenic mice expressing human CEACAM-1 [15]. NadA is another major adhesin that is encoded by many of the hypervirulent meningococcal lineages but is not universally distributed. The ligand for this adhesin is thought to be β1 integrins and/or LOX-1 [16, 17]. Further, NadA is a major component of the meningococcal group B vaccine Bexsero [18]. Several other OMPs have been shown to contribute to host cell adhesion (see [1, 9] and references therein). The relative contributions of the pilus and these OMPs to host cell adhesion is not clear and may vary between Nm lineages and host cell surfaces.

A critical potential regulator of Nm adhesion to host surfaces is localized hypermutation [19]. In the majority of Nm strains, the major pilin subunit, PilE, is subject to high-frequency antigenic variation involving recombination between the *pilE* expression locus and non-expressed *pilS* loci [20]. Similarly, comprehensive genome analyses have detected a wide distribution of other adhesins with the potential to undergo phase variation (PV) mediated by high frequency, reversible insertions/deletions within simple sequence repeat (SSR) tracts [21]. Mutations in these repetitive sequences can produce frameshifts, switching expression ON/OFF, or modulate promoter activity between high, medium and low levels of gene expression [22]. The Opa and PilC proteins are subject to PV due to pentanucleotide (5′CTCTT) or mononucleotide (5′C) repeats, respectively, present in the reading frame, while PV of NadA is mediated by a 5′TAAA repeat located upstream of the promoter region [23]. Localized hypermutation results in heterogeneous populations of meningococci in the nasopharynx expressing different combinations of these phase-variable PV adhesins [24, 25]. This heterogeneity is likely to result in isolates having differing abilities to adhere to and disrupt the host epithelium. PV may also facilitate cycling between host adhesion and escape of non-specific and antigenic-specific immune responses. While the roles of these hypermutable adhesins have been analysed in laboratory-passaged disease strains using mutants and, in some cases, phase variants, there have been few comparative studies utilizing multiple natural isolates from asymptomatic human carriers.

The human lung adenocarcinoma cell line, Calu-3, has an epithelial cell morphology and has been widely used for studies of meningococcal adhesion phenotypes. These cells form a polarized monolayer when grown with an air interface, with formation of strong tight junctions resulting in low permeability [26]. Previous studies with this cell line have detected efficient adhesion, microcolony formation, cellular remodelling and transcellular passage by *N. meningitidis* strains 8013 and MC58 [27–29]. We utilized this model system to investigate the capacity of carriage isolates of the MenY:cc23, MenY:cc174 and hypervirulent MenW:cc11 lineages to alter monolayer permeability and stability. In addition, we investigated how localized hypermutation of the OMPs encoded by *nadA*, *pilE* and the multi-copy *opa* loci influence interactions of meningococci with host epithelial cells. Our studies have revealed evidence of variability in the ability of meningococcal isolates to adhere to, invade and disrupt epithelial cell layers, and the contributions of some of the key adhesion molecules.

## Methods

### Meningococcal strains and growth conditions

Carriage isolates were obtained from nasopharyngeal swabs from University of Nottingham students. The MenW:cc11 carriage strains (R001, B285 and R191; Table 1) were obtained from a cross-sectional study conducted between September 2015 and March 2016 [30]. The MenY:cc174 (N59.1) and MenY:cc23 (N222.1, N459.3 and N459.6) carriage strains were obtained from a longitudinal study between October 2008 and May 2009 [31]. Strains were subject to whole-genome sequencing, and genogrouping to determine capsule type and clonal lineage [32, 33]. The NadA peptide of each strain was determined by querying the gene sequence using the PubMLST *Neisseria* database [34]. Expression states of phase-variable genes were previously determined by PCR-based fragment size analysis and sequence translation, and confirmed for sub-sets of isolates by immunoblotting [35, 36]. Meningococcal strains were cultured on brain heart infusion (BHI) agar plates supplemented with 5 % horse blood (Oxoid, UK) at 37 °C in the presence of 5 % CO_2_.

**Table 1.** MenW:cc11 bacterial strains used in this study and their OMP expression states

Strain name	Geno*	CC/ST*	*nadA*†	opaA‡	o*paB*	o*paD*	o*paJ*	***opc*** §	*mspA*	*nalP*	*pilC1*	*pilC2*
R001	w	11/11	Low	OFF	OFF	OFF	OFF	na	OFF	ON	ON	ON
B285	w	11/11	High	ON	OFF	ON	OFF	na	OFF	ON	OFF	ON
R191	w	11/11	Low	ON	OFF	ON	OFF	na	ON	ON	ON	ON
N222.1	Y	23/1655	n/a	OFF	ON	OFF	OFF	High	OFF	OFF	ON	OFF
N459.3	Y	23/1655	n/a	ON	OFF	OFF	ON	Low	OFF	OFF	OFF	ON
N459.6	Y	23/1655	n/a	OFF	OFF	OFF	ON	Low	OFF	OFF	ON	OFF
N59.1	Y	174/1466	High	ON	OFF	OFF	OFF	Low	OFF	ON	OFF	ON

^∗^Geno, genogroup; ST, sequence type; CC; clonal complex.

†*nadA* expression is classified into low, intermediate/high; *nadA* 5’TAAA repeat numbers: R001, 12; B285, 11; and R191, 9, as reported by Green *et al.* [35]; MenW strains have peptide variant 2/3; *nadA* is absent in cc23 strains (na, not applicable).

‡opaA alleles differed between the three MenW:cc11 isolates, with R001, B285 and R191 having alleles 2, 1 and 2, respectively (Fig. S1).

§Opc expression is classified into low, intermediate and high, as reported in [24]; *opc* is absent in the MenW:cc11 strains.

### Generation of pilE/opa/nadA mutants

All primers used for construction and confirmation of mutants in this study are listed in Table S1 (available in the online version of this article). The *pilE* gene and ~200 bp upstream and downstream were amplified using primer pairs PilEMut_US_F/ PilEMut_US_R and PilEMut_DS_F/PilEMut_DS_R from R001, B285 and R191, respectively. Approximately 200 bp of the *pilE* sequence was replaced with an erythromycin resistance cassette, amplified using PilEMut_Ery_F/PilEMut_Ery_R from pDH2O. For the *opa* mutants, a single generic *opa* construct was generated that would insert into all of the individual *opa* alleles in the three wild-type (WT) strains. Two 200 bp fragments of the *opa* gene sequence were amplified using primer pairs OpaMut_US_F/OpaMut_US_R and OpaMut_DS_F/OpaMut_DS_R, respectively. A kanamycin cassette was amplified from pJMK30 using OpaMut_Kan_F/OpaMut_Kan_R and inserted between these fragments, resulting in 180 bp *opa* sequence deletion. All PCR amplifications were performed using Q5 High-Fidelity DNA Polymerase (NEB, USA) following the manufacturer’s instructions. Gene and flanking sequences for both *pilE* and *opa* constructs were assembled using the NEBuilder HiFi DNA assembly master mix (NEB, USA) following the manufacturer’s instructions. Each purified construct was naturally transformed into each strain as described previously [37] and single colonies were selected for on BHI agar plates supplemented with either 50 µg ml^−1^ kanamycin or 5 µg ml^−1^ erythromycin. Disruption of a specific *opa* allele was confirmed by PCR amplification using primers specific for the flanking regions of each individual *opa* locus (Table S1). The *nadA* construct used in this study has been previously described [35].

Immunoblot confirmation of protein expression was performed as previously described [35]. Briefly, bacterial suspensions of relevant strains grown on agar plates were prepared in phosphate-buffered saline (PBS) to an OD_600_ of 0.5. Meningococcal cells were pelleted from a 1.5 ml aliquot by centrifugation at 13 000 r.p.m. and resuspended in sodium dodecyl sulfate (SDS) loading buffer. Suspensions were heated for 10 min at 100 °C. Aliquots were separated on a 10 % SDS polyacrylamide gels and transferred to polyvinylidene difluoride (PVDF) membranes for Western blotting. Blots were either probed with a pilin class II-specific antibody (EP112606, 1 : 3000 kindly gifted by Christoph Tang, Oxford [38]) followed by an anti-rabbit HRP conjugate (Sigma Aldrich, UK, 1 : 4000), or with a cross-reactive Opa antiserum (Al-Rubaiawi and Bayliss, unpublished data) followed by an anti-mouse HRP conjugate (Thermo Fisher, USA, 1 : 2000). The reaction was detected using EZ-Chemiluminescence (Geneflow, UK) and photographic film.

### Cell lines and culture conditions

The adenocarcinoma A594 (ATCC CCL185) and Calu-3 (ATCC HTB-55) human lung epithelial cell lines were maintained in RPMI-1640 (Gibco, UK) media supplemented with 10 % foetal bovine serum (FBS; Gibco, UK) and in Dulbecco’s modified Eagle’s medium/Nutrient Mixture F-12 Hams (1 : 1; Gibco, UK) with 10 % FBS, respectively. Both cell lines were maintained at 37 °C in the presence of 5 % CO_2_. A549 cells were routinely cultured in T75 flasks and maintained at ~70 % confluence. Calu-3 cells were cultured at the air–liquid interface (ALI) in 12-well 0.4 µm pore size transwell insert plates (Corning, USA) and the apical side of each insert was seeded at a density of 1×10^5^ cells in 0.5 ml medium with 1 ml of medium in the basolateral chamber. The cells were incubated overnight and medium from the apical chamber was aspirated. Medium in the basolateral chamber was replaced every 2 days. Cells were left to differentiate for 21 days before experiments were performed.

### Bacterial adhesion and internalization assays

Single colonies of R001, B285 and R191 and their relevant mutants were cultured in BHI broth at 37 °C in the presence of 5 % CO_2_ with shaking overnight. After incubation, cultures were diluted and grown to mid-log (OD_600_=0.4) and 1×10^5^ A594 human cells were seeded into 24-well plates on the day prior to an assay. Cells were infected at a multiplicity of infection (m.o.i.) of 30 for 1 h. After removal of the bacterial inoculum, cells were gently washed with PBS followed by the addition of RPMI-1640 and incubation for 18 h. After incubation, non-adherent bacteria were removed by washing three times with PBS. To detect bacterial adhesion, cells were lysed with 0.1 % Saponin (Sigma, UK) for 20 min followed by plating of serial dilutions of the lysates on agar plates to enumerate the colony-forming units (c.f.u.). To detect internalization, cells were incubated with 100 µg ml^−1^ gentamicin in RPMI-1640 for 30 min followed by cell lysis with 0.1 % Saponin (Sigma, UK). An identical method was utilized for assays with Calu-3 assays, except that cells were cultured on 0.4 µm pore size transwell polyester insert plates (Corning, USA).

### Transepithelial electrical resistance (TEER) measurements

TEER measurements were performed on Calu-3 monolayers growing on transwell inserts using a Millicell ERS-2 (Millipore, USA) according to the manufacturer’s instructions. TEER was measured at 21 days post-seeding. Wells containing medium and transwells without Calu-3 cells were measured as blank controls. TEER was calculated using the following formula:

,$$TEER\;\left(\Omega \;cm^{2}\right)=\left(S-B\right)x\;MA\;\left(1.12\;cm^{2}\right)$$

where *S* is sample resistance, *B* is blank resistance and *MA* is the membrane area.

A TEER (Ωcm^2^) measurement of >400Ωcm^2^ was considered to represent a confluent monolayer with intact tight junctions [39].

### FITC permeability assay

Calu-3 cells were initially seeded and differentiated as for bacterial adhesion assays. Monolayers were infected with bacteria (m.o.i. 30) as described above with DMEM/F12 medium in the basolateral chamber. After 18 h incubation, cells were washed three times with PBS. Hank’s balanced salt solution (HBSS) (Gibco, UK) supplemented with 25 mM HEPES (Sigma, UK) and 1 μg mL^−1^ of fluorescein isothiocyanate (FITC)-dextran 10-kD (Sigma, UK) was added to the apical chamber, and HBSS supplemented with 25 mM HEPES without FITCdextran was added to the basolateral chamber. Aliquots were collected from the basolateral chamber every 30 min for 3 h and placed into a black flat bottom 96-well plates (Corning, UK). FITC-dextran was detected using an Omega FLUOstar plate reader (BMG Labtech, UK) with excitation and emission wavelengths of 485 and 520 nm, respectively. FITC-dextran was serially diluted in HBSS with 25 mM HEPES in the same plate and used to generate a standard curve. Concentrations in test wells were calculated by comparison of raw fluorescence readings to this standard curve.

### GeneScan analysis of PV gene expression states

To determine the PV expression state of *opa* alleles before, and after, adhesion and invasion assays a multiplex PCR that amplifies all four *opa* alleles was performed using primer pairs Opa_Con_F and Opa_Con_R (Table S1). Primer Opa_Con_F was fluorescently labelled with 6-FAM to allow fragment size analysis on an ABI3031xl genetic analyser (Applied Biosystems) using GeneScan 600 LIZ dye (Thermo Fisher, UK) as a size standard. The fragment sizes were determined and converted into repeat numbers using Peak Scanner (v 2.0). The same analysis was conducted for *nadA*, *mspA* and *nalP* using primer pairs previously described [24].

### Immunofluorescence staining, confocal microscopy and image analysis

For immunofluorescence staining of A549 cells, infection assays were performed in glass chamber slides (Nunc Lab-Tek, USA). Post-infection (p.i.), cells were fixed with 4 % EM-grade formaldehyde (Sigma Aldrich, USA) for 20 min at room temperature. The cells were washed three times in PBS and incubated for 10 min with 0.1 % Triton-X 100 (Sigma Aldrich, USA) to permeabilize the cells. The cells were blocked with PBS/5 % goat serum (blocking solution) for 30 min and then probed with a polyclonal anti-* N. meningitidis* antibody (Abcam, UK; 1 : 100) in blocking solution for 1 h. Following removal of unbound antibody by washing three times with PBS, cell monolayers were probed with alexafluor488-conjugated anti-rabbit secondary antibody (Invitrogen, USA; 1 : 500) in blocking solution and alexafluor647-conjugated phalloidin (Invitrogen, USA; 1 : 1000) in blocking solution for 45 min. After incubation, cells were washed three times in PBS and stained with DAPI (Invitrogen, USA). The slides were mounted with anti-fade diamond (Thermo Fisher, USA) and stored in the dark at 4 °C. For Calu-3 monolayers, the cells were fixed with ice-cold methanol and the membrane was cut out from transwell inserts and placed into a new 12-well plate for staining. Cells were permeabilized with 0.1 % saponin (Sigma, UK) and the staining protocol was carried out as above.

Images were visualized using an Olympus FV1000 confocal laser scanning microscope with a 60× objective. For visualization and quantitative analysis of A549 assays, we used the image analysis platform Fiji [40]. For each experimental condition at least 30 cells were analysed from at least 15 random fields of view. To determine the number of cell-associated bacteria in fluorescence images, regions of interest (ROI) encompassing single cells were derived using brightfield images. These ROIs were then imposed onto the bacterial fluorescence channel, and the number of bacteria were determined using the analyse particles function.

### Statistical analysis

Adhesion, invasion and permeability assay values were analysed by analysis of variance (ANOVA) or chi-squared tests in GraphPad Prism. A *P* value of <0.05 was considered significant.

## Results

### Comparison of adhesion in A549 and Calu-3 cells for carriage isolates of MenW:cc11, MenY:cc23 and MenY:cc174 lineages

Adhesion to epithelial cell surfaces is a critical initial step in host colonization by invasive meningococcal strains. The ability of carriage isolates of the MenW:cc11, MenY:cc23 and MenY:cc174 lineages to attach to epithelial cells was assessed by measuring adherent bacterial cells after 18 to 24 h incubation with immortalized epithelial cells. The MenY:cc174 isolate (N59.1) was utilized in these assays as a control, as this clonal complex is rarely associated with IMD. The adherence capacity of N222.1, a MenY:cc23 carriage isolate, and two derivatives (N459.3 and N459.6) obtained from the same carrier but after 6 months carriage, was examined on semi-confluent monolayers [25]. These three isolates are all variants of the same strain that has undergone adaptation during persistence in a single carrier [24, 25]. All three isolates exhibited similar levels of adhesion to semi-confluent A549 and Calu-3 monolayers after both 6 and 24 h incubation (Fig. S2).

**Fig. 1.**
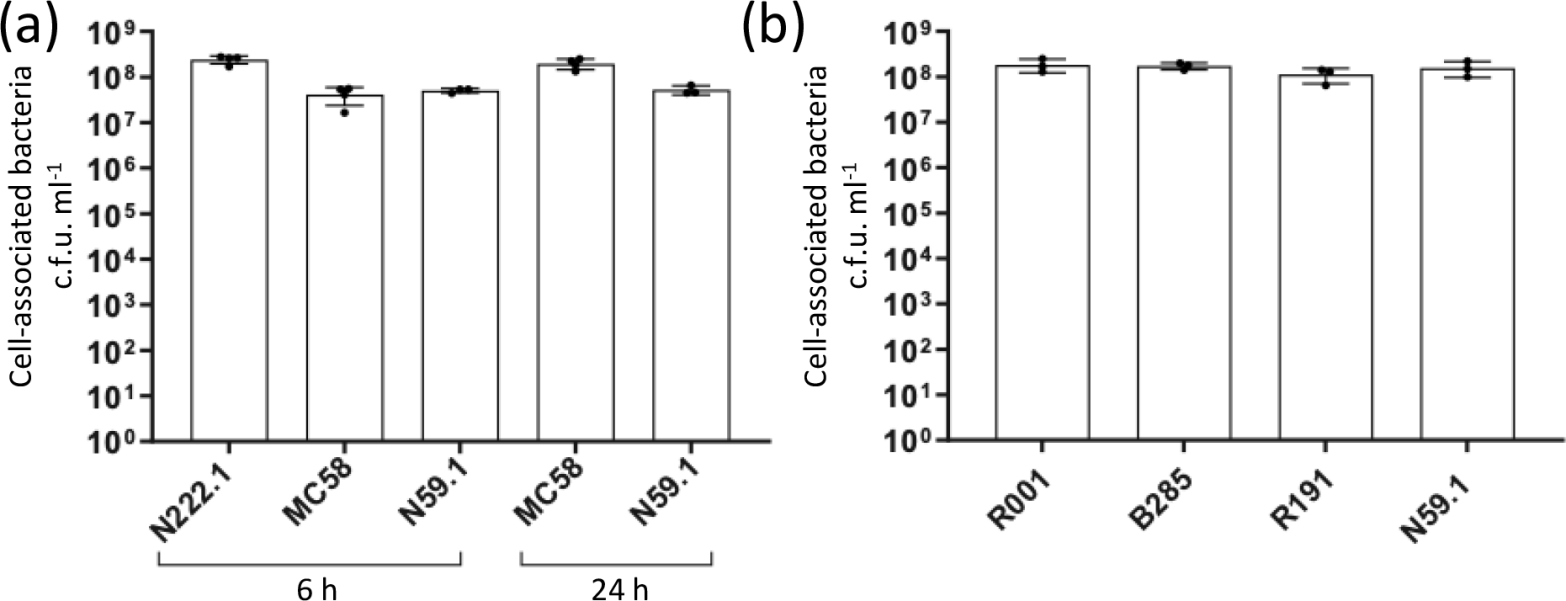
Adherence of carriage isolates of the MenY:cc23 and MenW:cc11 lineages to Calu-3 monolayers grown in an air–liquid interface. (**a**) Calu-3 cells were infected with N222.1, MC58 and N59.1, MenY:cc23, MenB:cc32 and MenY:cc174 isolates, respectively, at an m.o.i. of 30 for 6 and 24 h. No data were obtained for N222.1 at 24 h due to disruption of the monolayers. (**b**) Calu-3 monolayers were infected with three MenW:cc11 isolates (i.e. R001, B285 and R191) at an m.o.i. of 30 for 1 h before removal of bacteria, and replacement with medium and incubation for 18 h. After incubation, non-adherent bacteria were removed by sequential PBS washes and lysed with 0.1 % saponin to release cell-associated bacteria. Bacterial c.f.u. counts were determined by plating serial dilutions on BHI agar plates. Error bars represent the standard deviation of the mean of three replicates (MenY) or three independent experiments (MenW). No significant differences were detected by an ordinary one way-ANOVA.

The adherence capacity of the N222.1 and N59.1 isolates were then tested on ALI Calu-3 monolayers, with both isolates exhibiting comparable levels of adherence after 6 h incubation (Fig. 1a). At 24 h, high levels of adhesion by N59.1 were detected (Fig. 1a), but monolayers infected with N222.1 were completely disrupted, preventing measurements of levels of adhesion. An additional control, MC58, a MenB:cc32 disease isolate utilized in previous ALI experiments, was included in these experiments and exhibited similar levels of adherence to the N59.1 strain at 6 and 24 hours p.i. (Fig. 1). All strains showed similar levels of replication in the supernatant, indicating that growth in the tissue culture medium was not influencing the rates of adherence and invasion (Fig. S3).

Three isolates were selected for the MenW:cc11 lineage that were known to differ in their NadA or Opa gene PV expression states (Table 1). In initial experiments, significant levels of cytotoxicity were observed after 24 h with these isolates (data not shown). Infections were modified with removal of the inoculum after 1 h followed by incubation for a further 18 h. No significant differences in cell-associated bacteria were observed between the three MenW:cc11 isolates on either semi-confluent A549 monolayers (data not shown) or ALI Calu-3 cells. For the N222.1 MenY:cc23 and all MenW:cc11 isolates, ~1×10^8^ adherent bacteria were detected on ALI Calu-3 monolayers (Fig. 1).

### Differential disruption of Calu-3 monolayers by meningococcal carriage isolates

Disruption of epithelial barrier functions is a potential attribute leading to systemic spread of meningococci. Nm isolates were tested for their ability to disrupt monolayers of Calu-3 cells grown in ALI with the apical cell surface exposed to air, which mimics conditions in the upper respiratory tract. Permeabilization of infected ALI monolayers was examined by measuring the amounts of FITC-dextran (µg ml^−1^), which permeated from the apical to the basolateral medium over a 3 h time period in comparison to uninfected cells. For MenY isolates, infections were performed for 12 h without removal of the inoculum, with this time point selected due to the high levels of disruption observed for N222.1 in adhesion experiments at 24 hours p.i. (data not shown). MenW isolates also demonstrated high levels of monolayer disruption, requiring a change in methodology. Cells were infected for 1 h before bacteria were removed and the inoculum was replaced with fresh media. This was followed by incubation of the infected monolayers for 18 h prior to testing for permeabilization. The MenY:cc174 isolate, N59.1, was included in both experiments and data were normalized relative to this strain in order to facilitate comparisons between the two strain groups.

**Fig. 2.**
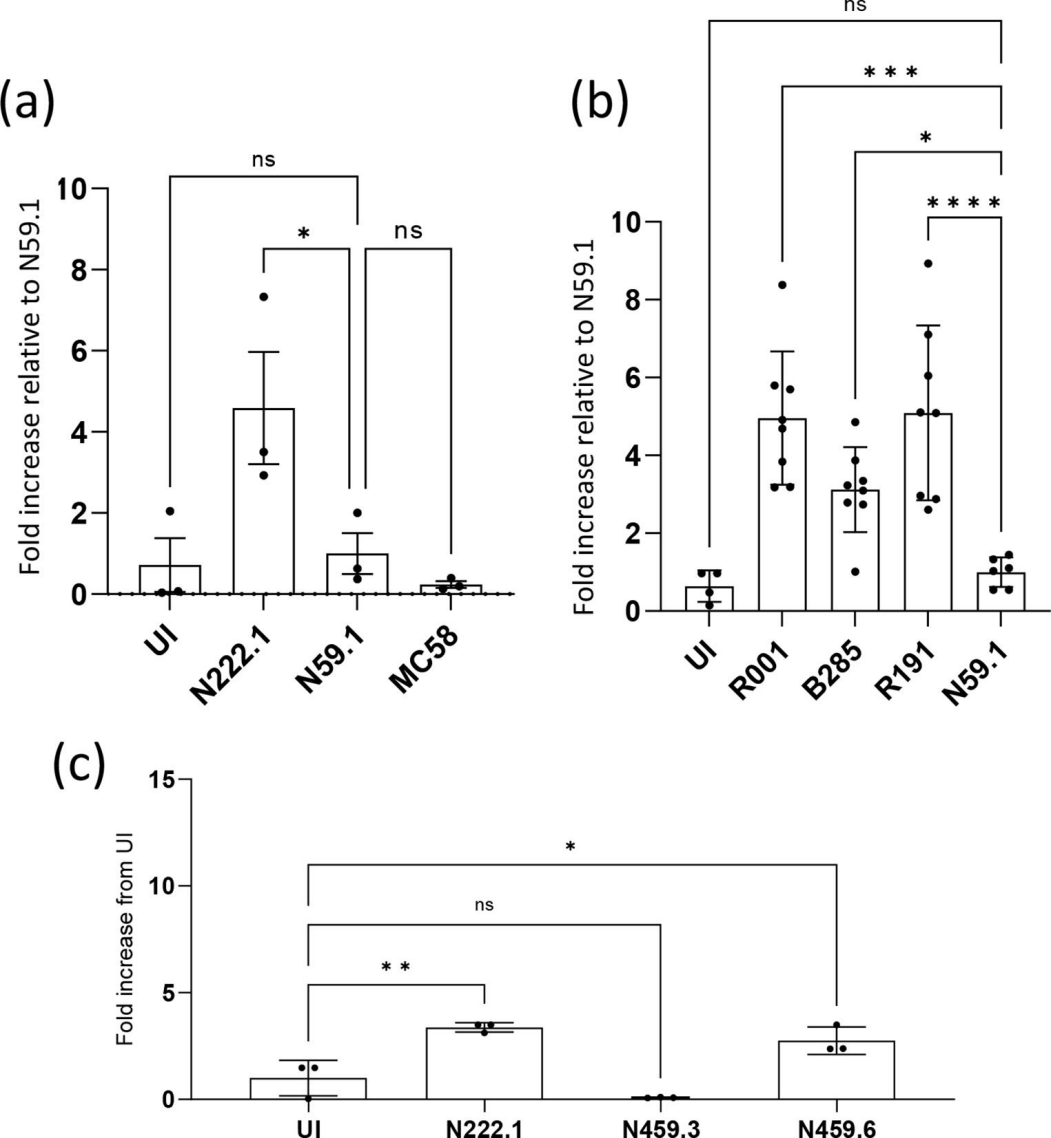
Effect of MenY and MenW:cc11 isolates on Calu-3 monolayer permeability. Calu-3 cells were grown in transwell plates under ALI conditions to confluent monolayers. Permeabilization of uninfected and infected cells was tested by adding FITC-dextran to the upper chamber and measuring the amounts of this molecule in the basolateral chamber after 3 h incubation. (**a**) Monolayers were infected at an m.o.i. of 30 with MenY:cc23 (N222.1), MenY:cc174 (N59.1) or MenB:cc32 (MC58) isolates for 12 h. (**b**) Monolayers were infected with three different MenW:cc11 (m.o.i. 30) isolates or N59.1 for 1 h followed by removal of the inoculum and incubation for 18 h. (**c**) Monolayers were infected with three MenY:cc23 isolates (N222.1, N459.3 and N459.6) from carrier V222 at an m.o.i. of 30 for 12 h. Levels of FITC-dextran in the basal chamber are expressed as a fold increase from uninfected (UI) control cells. For panels (a, b), these data were then normalized relative to the fold increase for N59.1. Error bars show the standard deviation of the mean from three or eight replicates (filled circles) for the MenY and MenW isolates, respectively. Individual experiments consisted of two or more technical replicates. Significance values above each column indicate statistical comparisons with N59.1 (a, b) or the uninfected control (c). Significant differences are indicated: ns, not significant; *, *P*<0.05; and ***, *P*<0.001 (ordinary one way-ANOVA).

The N222.1 MenY:cc23 isolate was examined in comparison to the MenY:cc174 isolate and to MenB:MC58 (Fig. 2a). Permeabilization by N59.1 was very low and not significantly different to either uninfected cells or to cells infected with MC58 (Fig. 1a). The N222.1 isolate exhibited significant monolayer permeabilization relative to N59.1. In a separate experiment, the three MenY cc23 isolates from the same carrier were compared to each other (Fig. 2c). Significant heterogeneity in monolayer permeabilization was observed, with N222.1-infected cells displaying the highest level of FITC permeation and slightly lower levels for the N459.6 isolate (Fig. 2a, c). In contrast, both the N459.3 (Fig. 2c) and N59.1 (Fig. 2a) isolates exhibited low levels of permeabilization, similar to uninfected cells. While all three MenY cc23 isolates exhibited similar levels of adhesion to non-confluent Calu-3 cells, adhesion to ALI monolayers was not tested such that differences in disruption of the monolayers may have been due to differential levels of adhesion to the ALI Calu-3 monolayers rather than to a differential capacity to disrupt monolayers. Results with the MenW:cc11 lineage were more consistent, with all three isolates causing disruption of these monolayers (Fig. 2b).

### PilE is critical for effective adhesion of multiple MenW:cc11 to host epithelial cells

**Fig. 3.**
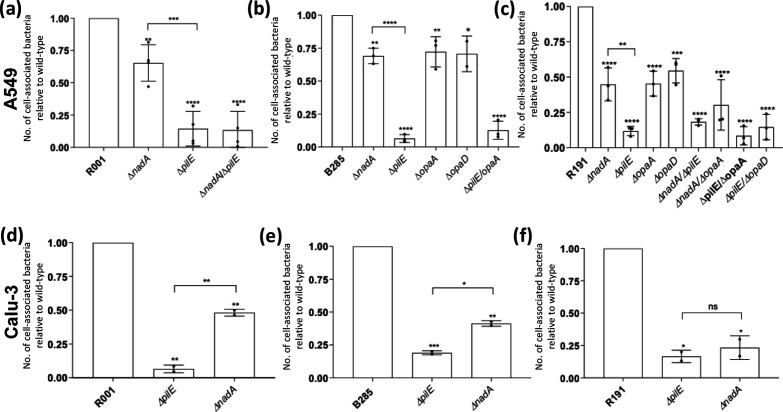
Effect of disruption of known adhesins on adherence of MenW:cc11 isolates to human epithelial cells. Semi-confluent A549 human epithelial cells (**a–c**) and ALI Calu-3 monolayers (**d–f**) were infected with wild-type or mutant meningococcal strains at an m.o.i. of 30 for 1 h followed by removal and replacement of the inoculum with media and incubation for 18–22 h, respectively. After incubation, non-adherent bacteria were removed by sequential PBS washes followed by lysis of the cells with 0.1 % saponin and enumeration of bacterial cells by plating of serial dilutions on agar plates. Error bars show the standard deviation of the mean from three independent biological replicates (filled circles). Individual experiments consisted of two or more technical replicates. Significance values above each column indicate statistical comparisons with the relevant wild-type strain. Comparisons between columns are shown by connecting lines. Significant differences are indicated: ns, not significant; *, *P*<0.05; **, *P*<0.01 ***, *P*<0.001; and ****, *P*<0.0001 (ordinary one way-ANOVA).

In order to determine whether major meningococcal adhesins were involved in disruption of epithelial monolayers, deletion mutations were constructed in the *nadA*, *pilE* and *opa* loci of the three MenW:cc11 isolates. These mutants were tested for adhesion to two human epithelial cell lines grown under different conditions: semi-confluent A549 cells (Fig. 3a–c) and ALI Calu-3 monolayers (Fig. 3d–f). Deletion of *pilE* in all three strain backgrounds led to a significant, but not completely ablated, reduction in adherent bacteria for both cell lines. Deletion of *nadA* also produced significant, but minor, reductions in adherence to both cell types. These reduced levels of adherent bacteria were, however, significantly higher than for the *pilE* mutants in all strains except for R191 with ALI Calu-3 monolayers. Deletion mutants were constructed for *opaA* and *opaD* in the B285 and R191 strains where these genes are in ON PV states (Table 1). These deletions conferred a modest reduction in adhesion to the A549 cells (Fig. 3a). Deletion of either *nadA* or *opa,* together with *pilE*, conferred no additional reduction in adhesion compared with the *pilE* mutant alone (Fig. 3a), indicating a dominant role for PilE in host cell attachment. All mutants were tested for rates of replication in the monolayer tissue culture media and exhibited similar growth curves to the respective WT strain, indicating that differences in adhesion were not due to differing abilities to replicate in the tissue culture media (Fig. S4).

### Dominant role for *nadA* in invasion of host epithelial cells

**Fig. 4.**
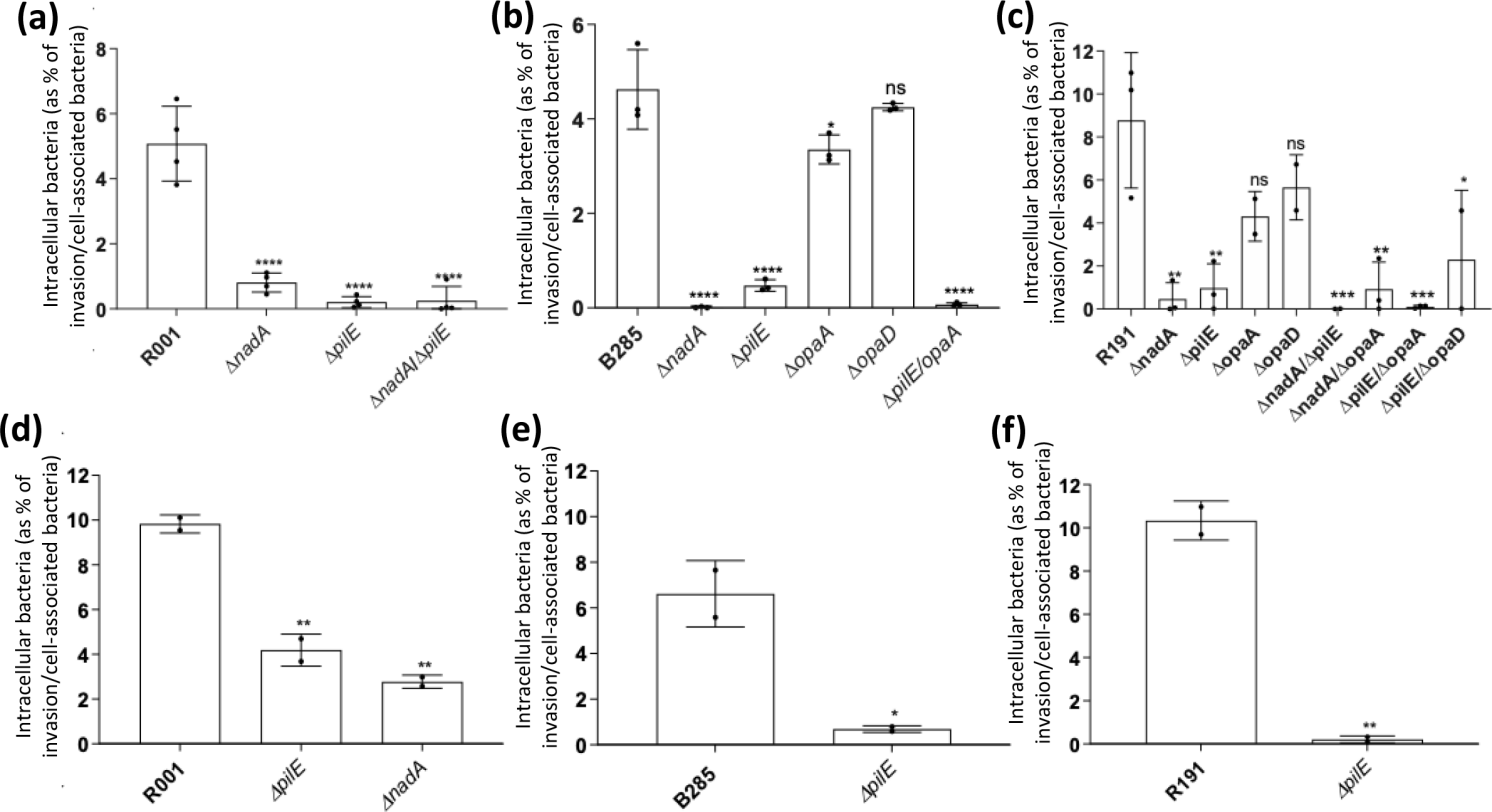
Effect of deletion of adhesins on invasion of human epithelial cells by MenW:cc11 strains. Initial infections were performed as described in Fig. 3. Numbers of viable intracellular bacteria were determined by subjecting infected cells to a 30 min incubation with 100 µg ml^−1^ gentamicin in order to kill extracellular and adherent bacteria. After removal of the antibiotic, intracellular bacteria were released by lysis of the eukaryotic cells with 0.1 % saponin and enumerated. Invasion c.f.u. counts were normalized against the total number of cell-associated c.f.u. as determined prior to gentamicin treatment (see Fig. 3). Data are shown for A549 (**a–c**) and Calu-3 (**d–f**) cells with wild-type and mutant strains of R001 (**a, d**), B285 (**b, e**) and R191 (**c, f**). Error bars show the standard deviation of the mean from three independent biological replicates. Individual experiments consisted of two or more technical replicates. Statistical significance for each mutant compared to their respective wild-type strain are displayed on the graph. Significant differences are indicated: ns, non-significant; *, *P*<0.05; **, *P*<0.01 ***, *P*<0.001; and ****, *P*<0.0001 (ordinary one way-ANOVA for A549 and unpaired *t*-test for Calu-3).

To investigate the role of outer-membrane proteins in invasion of human epithelial cells, gentamicin was utilized to kill extracellular bacteria after 18–22 h of infection (Fig. 4). As adhesion is a prerequisite for invasion, the numbers of intracellular bacteria were normalized relative to the number of total cell-associated bacteria. In contrast with the adhesion data, deletion of *nadA* conferred a large and highly significant reduction in invasion by all three MenW strains in the A549 cells and by the R191 strain in ALI Calu-3 monolayers. A highly significant reduction in invasion was also observed with the *pilE* deletion across all three strains in both cell lines. This suggests that the pilus is not only involved in adherence but plays a major role in meningococcal invasion. Deletion of the *opa* genes did not produce significant reductions in the invasion of A549 cells. These outputs suggest that NadA, but not Opa proteins, play a major role in entry to host cells.

### Disruption of NadA and Opa alters the formation but not size of microcolonies

**Fig. 5.**
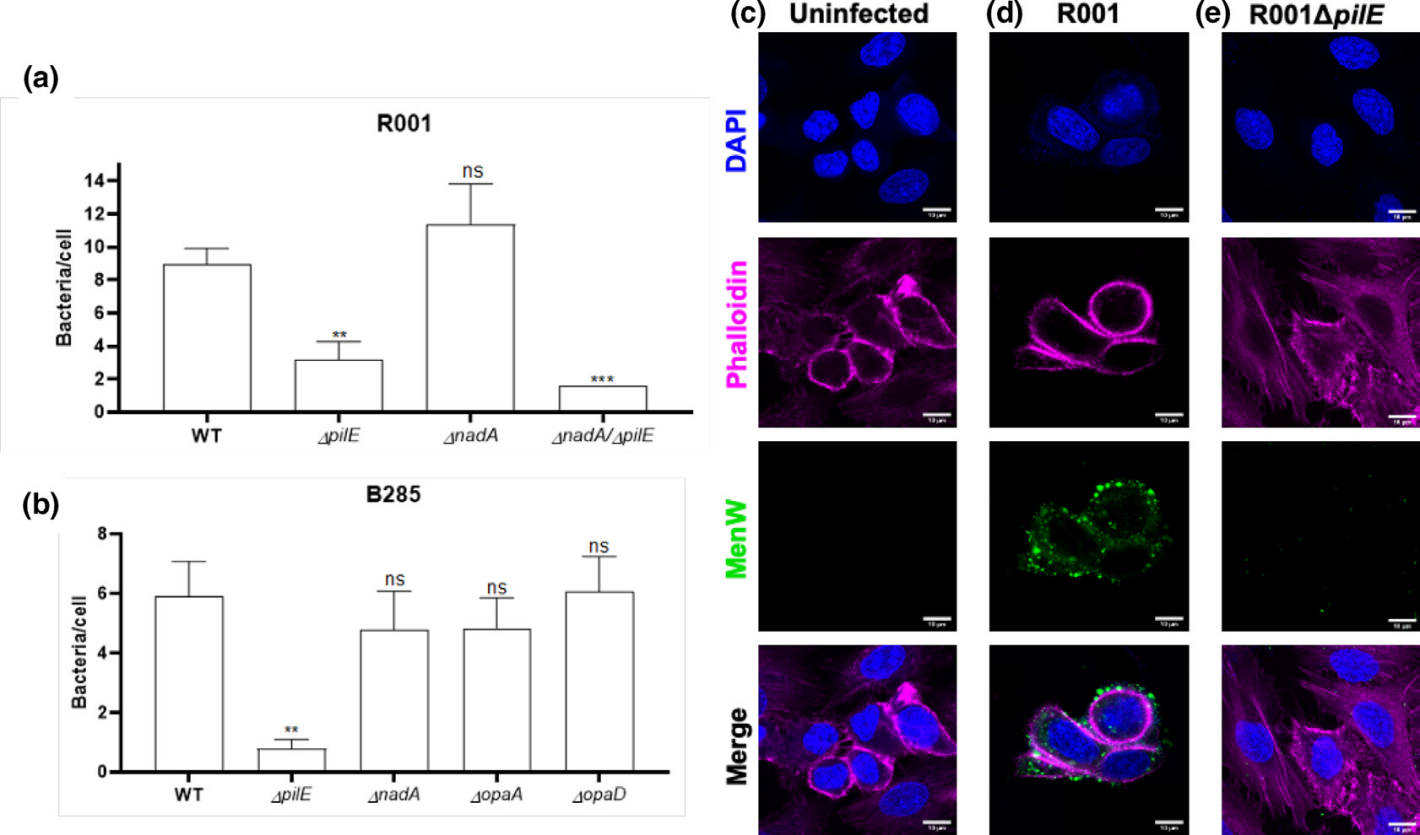
Confocal microscopy analysis of meningococcal adherence to A549 cells. Microcolony formation on A549 cells was analysed after 19 h of infection for the R001 and B285 strains [(a, b), respectively]. Adherent meningococcal cells were detected utilizing a polyclonal *N. meningitidis* antibody and visualized by confocal microscopy. The numbers of meningococcal cells per individual A549 cell were quantified using the Fiji program. The data represent the mean and standard error (see Fig. S5 for individual data points). Statistical significance was tested for each mutant as compared to their respective wild-type strain. Significant differences are indicated: *, *P*<0.05; **, *P*<0.01; and ***, *P*<0.001 (one-way ANOVA). Exemplar confocal microscopy images of uninfected cells (**c**) and cells infected with either R001 (**d**) or R001*ΔpilE* (**e**) are shown. Nuclei are stained with DAPI (blue) and actin with a phalloidin–alexafluor-647 conjugate (magenta). Meningococci were detected with a polyclonal *N. meningitidis* antibody followed by alexafluor488-conjugated secondary antibody (green). Scale bars, 10 um. Channels from each individual fluorophore are displayed in addition to a merged image for each condition.

Aggregates of bacterial cells, referred to as microcolonies, are a significant feature of meningococcal attachment to host cells. Confocal microscopy was utilized to further analyse the contributions of the major meningococcal adhesins to attachment to host cells and to evaluate the formation of microcolonies on these surfaces (Fig. 5). In this context, a microcolony was defined as the presence of five or more Nm cells present on one host cell. Microscopy was performed following infections of semi-confluent monolayers of A549 cells in order to allow for accurate designation of the numbers of bacteria attached to individual host cells. Infected cells were readily detectable, as shown in an exemplar microscopy image for strain R001 (Fig. 5d) as compared to uninfected cells (Fig. 5c). Both the B285 and R001 (Fig. 5a, b, respectively) WT strains exhibited high infection rates with very few uninfected cells and a mean number of bacteria per cell of nine and six for R001 and B285, respectively. Deletion of *pilE* resulted in the majority of cells remaining uninfected, with a significantly lower mean number of cell associated bacteria compared with the WT (Fig. 5a, b) and as exemplified for R001Δ*pilE* (Fig. 5e). Interestingly, in cases where cell-associated *pilE* mutant meningococcal cells were detected, the microcolonies were of comparable numbers to the WT (Fig. S5). This implies that deletion of the pilus leads to a lower probability of adhesion to host cells but does not disrupt microcolony formation. Statistically significant reductions in host cell colonization were not detected by this method for deletion mutants of the *nadA, opaA* or *opaD* genes. However, high numbers of non-infected cells were observed for these mutants with this reduced level of general adhesion compensated for by a non-significant increase in larger microcolonies (Fig. S5). Strain R191 was excluded from our analysis, as most A549 cells were lost during fixation, indicating that the surface attachment of these cells had been perturbed. These data indicate that the *nadA* and *opa* mutants may be defective for formation of microcolonies but retain the ability to form large aggregates on host cells.

### Adherence in the absence of PilE expression selects for phase-variable *opaD* expression

**Fig. 6.**
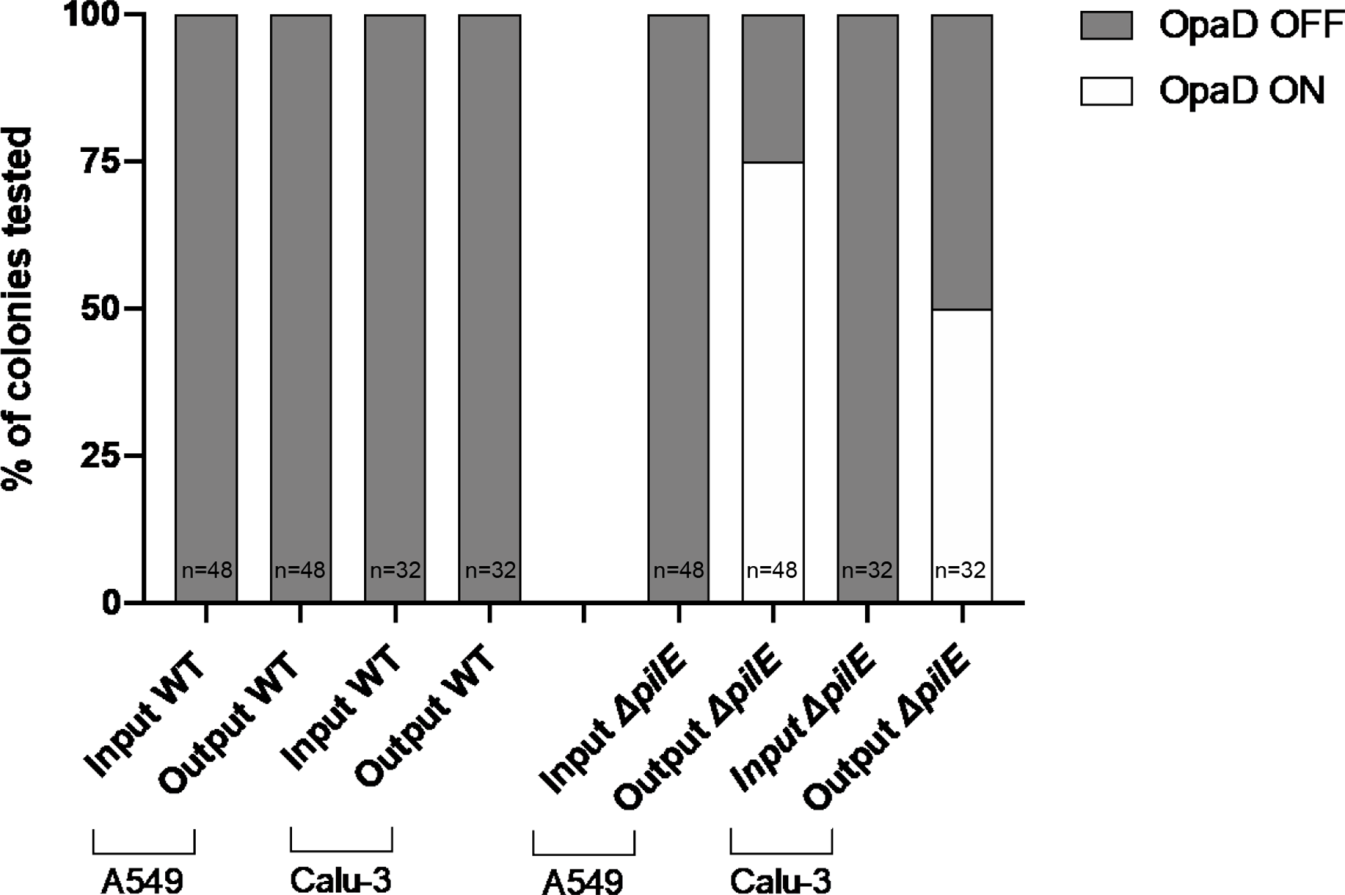
Expression states of *opaD* before and after infection of A549, and Calu-3 cells with wild-type R001 and R001*ΔpilE*. Opa expression states of input and output colonies from adhesion assays with wild-type R001 and R001*ΔpilE* in adhesion assays with A549 and ALI Calu-3 cells (see Fig. 3) were tested by GeneScan PCR. The graph shows the percentage of these colonies in either the ON (white bar fraction) or OFF (grey fraction) expression state. Data shown were obtained across four independent experiments for the A549 cells and two independent experiments for the Calu-3 cells (*n*, total number of bacterial colonies analysed).

To determine if host cell adhesion selects for PV of *opa* genes, we analysed the PV gene expression states of input and output colonies from adhesion assays using a PCR and GeneScan approach. All four *opa* genes were found to be in the OFF state for the WT and mutant R001 input strains (Table 1 and data not shown). Infection with R001 WT resulted in isogenic output colonies with no detectable differences in *opa* expression on both cell lines. Contrastingly, 50–75 % of output colonies from the R001*ΔpilE* mutant in the adhesion assays with A549 and ALI Calu-3 cells exhibited a switch in the *opaD* repeat tract from 23 to 24 repeats and a change in expression from OFF to ON (Fig. 6). No switches were observed in the other *opa* genes that have 12, 12 and 8 5′CTCTT repeats. Furthermore, no Opa switching was observed for outputs colonies of B285 WT, B285*ΔpilE*, R191 WT and R191*ΔpilE*, which have two *opa* alleles switched ON (Table 1). PV analyses of NadA, MspA and NalP were also conducted but no changes in gene expression were detected.

### Disruption of epithelial monolayers requires *pilE* expression, and is associated with tight junction damage

**Fig. 7.**
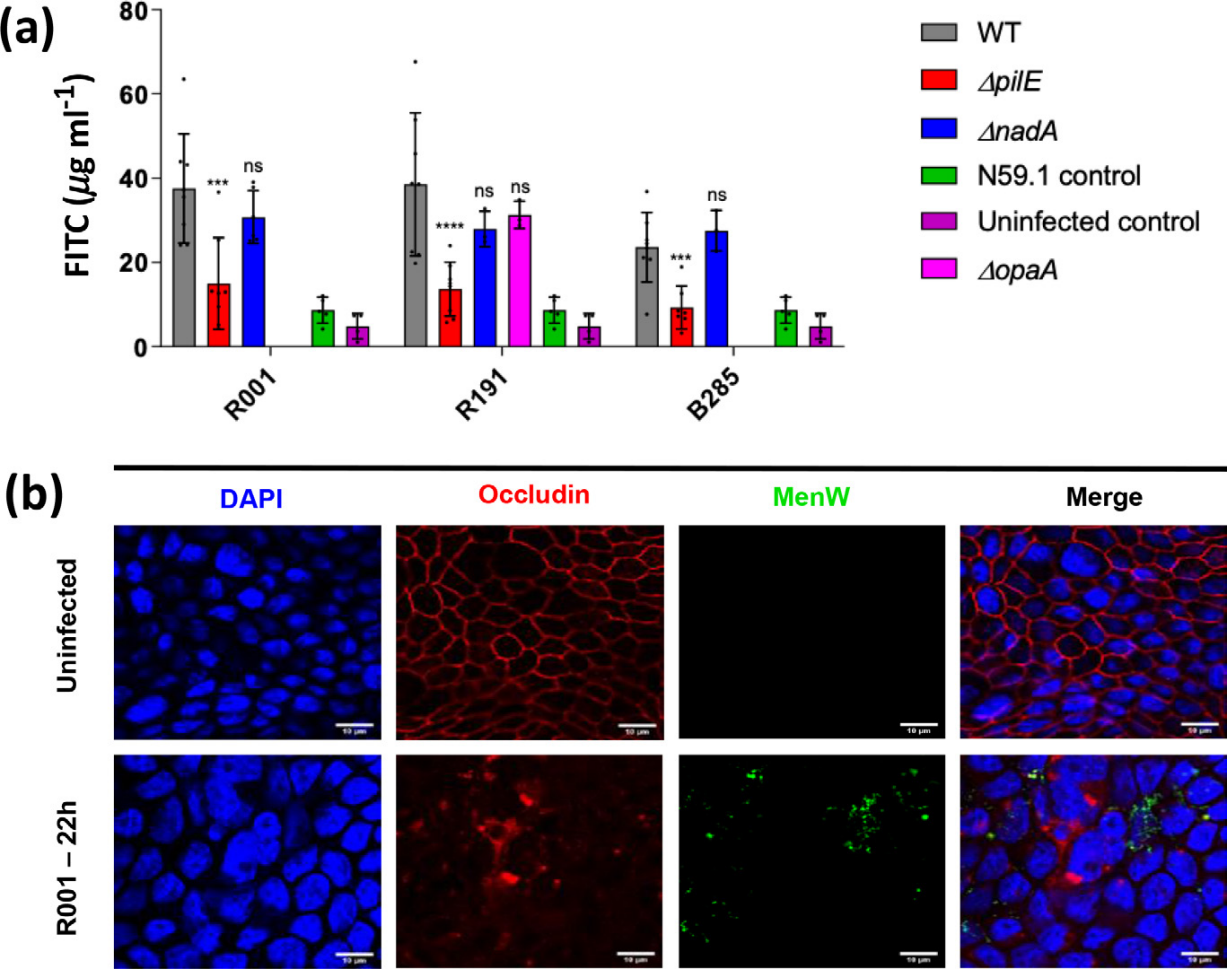
Effect of *pilE*, *opa* and *nadA* mutants of three MenW:cc11 strains on Calu-3 monolayer permeability and tight junction integrity. Monolayers of Calu-3 cells, formed in an air–liquid phase, were infected with MenW:cc11 R001 (m.o.i. 30) for 1 h followed by removal of non-attached bacteria and incubation for a further 18 h. (**a**) Monolayer permeability was measured by determining the quantity of FITC-dextran (µg ml^−1^) in the basolateral chamber after 3 h. Error bars show the standard deviation of the mean from at least three independent biological replicates. Each individual experiment was performed with technical triplicates. Significance values are for comparisons with the relative wild-type strain. *, *P*<0.05; **, *P*<0.01 ***, *P*<0.001; and ****, *P*<0.0001 (one way-ANOVA). The full-time course of FITC-dextran permeation can be found in Fig. S6. (**b**) Infected monolayers were fixed with methanol and stained using monoclonal antibodies specific for occludin or a polyclonal meningococcal antisera (*n*=4). Control, uninfected Calu-3 monolayer showed expected pattern of expression and localization for occludin. Calu-3 monolayers infected with *N. meningitidis* MenW:cc11 (green) at 22 h p.i showed loss of uniform occludin (red) staining and diffuse localization in the cytoplasm. Scale bars sizes are indicated in the figure.

Having established the relative contributions of the OMPs to host cell adhesion for the MenW:cc11 strains, we examined the impact of these OMPs on the integrity of monolayers formed in the ALI Calu-3 model. Permeabilization was tested for by measuring the amount of FITC-dextran that passed from the apical to the basolateral medium over a 3 h time period. A significant reduction in permeability was observed in the absence of *pilE* for all three strain backgrounds when compared to the WT strain (Fig. 7a; Fig. S6). No differences were observed for the *opa* and *nadA* mutants (Fig. 7a). Strain N59.1 (MenY:cc174) acted as a control and exhibited similar levels of permeability to uninfected cells (Fig. 7a).

Meningococci are known to disrupt tight junctions, and the presence and distribution of occludin were visualized by confocal microscopy. Uninfected Calu-3 monolayers showed uniform expression and localization of occludin (Fig. 7b). Infection with R001 resulted in reduced, uneven staining of the membrane and diffuse staining of the cytoplasm, indicating dissociation of occludin from the cell membrane, possibly by an MMP-8-mediated cleavage, as observed by Schubert-Unkmeir *et al.* [41] for meningococcal infections of human brain microvascular endothelial cell monolayers.

## Discussion

Meningococci are frequent, asymptomatic colonizers of the human nasopharynx, but some lineages have a higher propensity to traverse the respiratory epithelium and cause invasive infections [2, 5, 7]. The high genetic similarity of carriage and disease isolates indicates that disease-causing attributes are either inherent properties of disease-causing strains or arise due to rapid within-host adaptive processes. Disruption of monolayers is a phenotype that may contribute to progression from a carriage state to IMD. Our observations with the endemic MenY:cc23 and hypervirulent MenW:cc11 lineages indicate that independent carriage isolates can exhibit significant differences in disruption of epithelial monolayers and hence may differ in propensity for causing disease. Alternatively, isolates with reduced monolayer disruption may still traverse the epithelial barrier by transcytosis and hence still retain the capacity to cause disease.

Differing abilities to disrupt epithelial monolayers may be due to variable capacities to adhere to or invade host cells. Despite differences in *opa* PV expression states, similar levels of adherence to A549 and Calu-3 cells were exhibited by three MenY:cc23, one MenY:cc174 and three MenW:cc11 isolates, suggesting that adhesion or microcolony formation per se was not a determinant of epithelial disruption. Previous studies with MC58, a cc32 MenB strain, in this model, have demonstrated that the meningococcal type IV pilus is the dominant determinant of adhesion to and transcellular passage of Calu-3 monolayers in the absence of disruption of the barrier function [29]. Deletion of *pilE* in these MenW:cc11 strains reduced both adhesion to host cells and disruption of the epithelial monolayers. These findings indicate that the pilus is a key determinant of adhesion in this lineage and that adhesion or microcolony formation is required for disruption of the monolayers.

The NadA protein is confined to hypervirulent lineages but is nevertheless an important component of the MenB Bexsero vaccine [42]. The NadA-1 variant was previously shown to be a meningococcal invasin in the MC58 strain [43]. In the MenW:cc11 strains, we found that a NadA-2/3 variant plays a minor role in adhesion but contributes significantly to host cell invasion. All of the strains exhibited similar levels of invasion, even though NadA is in a ‘high’ PV expression state in the B285 strain compared to the other strains. This suggests that low/intermediate levels of NadA expression may be sufficient to mediate the invasion phenotype. Deletion of *nadA* perturbed host cell invasion but did not affect disruption of the epithelial monolayer, indicating that invasion of host cells is not required to initiate permeabilization of the monolayers or tight junction disruption.

The multi-copy Opa proteins enable adhesion to host epithelial surfaces via interactions with host cell CEACAM proteins and, for a sub-set of Opa Variants, HSPGs [1, 14]. Switching between Opa variants by PV has been observed during carriage [25] and is thought to facilitate continuous host cell adhesion in the presence of Opa variant-specific antibodies [44]. There are, however, limited data on the contributions of individual variants to host cell interactions and the impact of PV on these interactions. In our assays, high levels of adhesion, microcolony formation, invasion and monolayer disruption were observed for the R001 strain that has all the *opa* genes in an OFF state. No *opa* PV was seen with this WT strain, suggesting that the pilus is the major driver of these phenotypes. PV of the *opaD* gene, but not *nadA* or the autotransporters (i.e. *nalP* and *mspA*), was detected when the pilus was inactivated, indicating that OpaD can partially compensate for removal of the pilus for adhesion to these cell lines (Fig. 6). While we have not explored how microcolonies formed in our model systems, the observation of microcolonies for the pilus mutants may suggest that switching ON of the OpaD protein in these mutants enabled formation of bacterial aggregates prior to or post-adherence to the epithelial cells. Both the *opaA* and *opaD* genes were in an ON state in the other two MenW:cc11 strains. Deletion of these genes in isolation produced similar minor decreases in adhesion for A549 cells, while deletion in the context of the *pilE* mutant also resulted in comparably low levels of adhesion. The *opaA* and *opaD* genes within these MenW:cc11 strains have high levels of overall similarity but differ in the hypervariable regions (Fig. S1). Testing of binding to CEACAM-1-Fc proteins of the MenW cc11 isolates indicated that OpaD-expressing MenW cells had a lower level of binding to this ligand than OpaA-expressing cells, and hence that the OpaD protein might have an alternative ligand specificity [45]. Whether differential binding to CEACAM variants or HPSGs influences disruption of monolayers or adhesion phenotype in these strains requires further exploration. The observation of similar adhesive abilities indicates that PV between these variants could maintain adhesion while facilitating the potential for immune evasion.

The mechanistic basis for the differences between MenY:cc23 and MenW:cc11 carriage strains in their ability to disrupt epithelial monolayers is currently unclear. Retraction of the pilus facilitates intimate adhesion of meningococci to host cells, twitching motility and host cell remodelling, all of these phenotypes have the potential to contribute to tissue disruption [46–48]. Retraction of the meningococcal pilus is controlled by PilT but is also dependent on the PilC proteins, with the PilC1 protein being upregulated in a *pilT* mutant [49]. Both the PilC1 and PilC2 proteins are phase-variable and have divergent sequences indicative of differing functions. Morand *et al.* [50] observed differing cell type specificities for a MenC strain expressing either PilC1 or PilC2. The meningococcal isolates used in this study differ in their combinatorial PilC expression states (Table 1 [25]). The MenY isolates/strains with the highest (N222.1 and N459.6) and lowest (N459.3 and N59.1) level of monolayer disruption have different PilC phasotypes, with the former expressing PilC1 and the latter PilC2. These data suggest that the PilC genes may be determinants of the ability to disrupt host cells with switching from PilC1 to PilC2, as observed during persistent carriage of a range of MenY strains [25], reducing the ability to disrupt the epithelial barrier and hence also reducing the propensity to cause disease. However, the B285 MenW:cc11 isolate also expressed only PilC2, indicating that the specific PilC variant may be important for this phenotype.

In summary, our study demonstrates that three MenW:cc11 carriage strains and one MenY:cc23 carriage strain have the capacity to disrupt the monolayer integrity of Calu-3 cells grown in ALI conditions, whereas a cc174 carriage strain and one MenY:cc23 isolate lack this ability. We also show that the pilus, but not Opa or NadA proteins, is required for disruption of epithelial monolayers by the MenW:cc11 carriage strains. These findings mimic those of Coureuil *et al.* [46], who showed that strain 8013, a serotype C disease isolate, could disrupt monolayers of HCMEC/D3 endothelial cells in a pilus-dependent manner, and suggested that this phenotype may allow for paracellular passage across the blood–brain barrier. In contrast, other researchers have observed either the absence of or low level disruption of Calu-3 monolayers with MC58, a MenB disease isolate [25, 27]. Our findings indicate that a key phenotype with the potential to contribute to passage of Nm cells through the epithelial cell barrier, leading to invasive disease, may be widespread among Nm disease-causing lineages and specifically within the hypervirulent MenW:cc11 lineage. Further exploration of the mechanistic basis of this phenotype within and across Nm lineages and how strains regulate this phenotype has the potential to improve our understanding of the transition of meningococci from benign commensals to life-threatening disease-causing agents.

## Supplementary Data

Supplementary material 1
