# Supplementary material for: Variable disruption of epithelial monolayers by Neisseria meningitidis carriage isolates of the hypervirulent MenW cc11 and MenY cc23 lineages
Source: Microbiology (Reading). 2023 Feb 23;169(2):001305. doi: 10.1099/mic.0.001305 (PMC10197870; doi:10.1099/mic.0.001305)
Supplement: Supplementary material 1 [file mic-169-1305-s001.pdf]

```
opaJ               ATGAATCCAGCCCC-AAAAAACCTTCTCTTCTCTTCTCTTCTCTTCTCTTCCGCAGCGCA      59
opaA_Variant1      ATGAATCCAGCCCCCAAAAA-CCTTCTCTTCTCTTCTCTTCTCTTCTCTTCCGCAGCGCA      59
opaB               ATGAATCCAGCCCCCAAAAAACCTTCTCTTCTCTTCTCTTCTCTTCTCTTCCGCAGCGCA      60
opaD               ATGAATCCAGCCCCCAAAAAACCTTCTCTTCTCTTCTCTTCTCTTCTCTTCCGCAGCGCA      60
opaA_Variant2      ATGAATCcagCCCCCAAAAA-CCTTCTCTTCTCTTCTCTTCTCTTCTCTTCCGCAGCGCA      59
opaA_Variant3      ATGAATCCAGCCCCCAAAAA-CCTTCTCTTCTCTTCTCTTCTCTTCTCTTCCGCAGCGCA      59
                   ************** ***** ***************************************

opaJ               GGCGGCAAGTGAAGACGGCAGTCGCAGCCCGTATTATGTGCAGGCGGATTTAGCCTACGC      119
opaA_Variant1      GGCGGCAAGTGAAGACGGCAGCCGCAGCCCGTATTATGTGCAGGCGGATTTAGCCTACGC      119
opaB               GGCGGCAAGTGAAGACAGCGGGCACGGCCCGTATTATGTGCAGGCGGATTTAGCTTATGC      120
opaD               GGCGGCAAGTGAAGACAGCGGGCACGGCCCGTATTATGTGCAGGCGGATTTAGCTTATGC      120
opaA_Variant2      GGCGGCAAGTGAAGACGGCAGCCGCAGCCCGTATTATGTGCAGGCGGATTTAGCCTACGC      119
opaA_Variant3      GGCGGCAAGTGAAGACGGCAGCCGCAGCCCGTATTATGTGCAGGCGGATTTAGCCTACGC      119
                   **************** ** * * * **************************** ** **

opaJ               CGCCGAACGCATTACCCACGATTATCCGCAAGCAACCGGTGCAAA------CAACACAAG      173
opaA_Variant1      CGCCGAACGCATTACCCACGATTATCCGAAACCAACCGGTACAGACAAAGACAAAATAAG      179
opaB               CGCCGAACGTATTACCCACGATTATCCGAAAGCAACCGGTGCAAA------CAACACAAG      174
opaD               CGCCGAACGTATTACCCACGATTATCCGAAAGCAACCGGTGCAAA------CAACACAAG      174
opaA_Variant2      CGCCGAACGCATTACCCACGATTATCCGAAACCAACCGGTACAGACAAAGACAAAATAAG      179
opaA_Variant3      CGCCGAACGCATTACCCACGATTATCCGAAACCAACCGGTACAGACAAAGACAAAATAAG      179
                   ********* ****************** ** ******** ** *      *** * ***

opaJ               CACAGTAAGCGATTATTTCAGAAACATCCGTGCGCATTCCATCCACCCCCGGGTGTCGGT      233
opaA_Variant1      CACAGTAAGCGATTATTTCAGAAACATCCGTGCGCATTCCATCCACCCCCGGGTGTCGGT      239
opaB               CACAGTAAGCGATTATTTCAGAAACATCCGTGCGCATTCCATCCACCCCCGGGTGTCGGT      234
opaD               CACAGTAAGCGATTATTTCAGAAACATCCGTGCGCATTCCATCCACCCCCGGGTGTCGGT      234
opaA_Variant2      CACAGTAAGCGATTATTTCAGAAACATCCGTGCGCATTCCATCCACCCCCGGGTGTCGGT      239
opaA_Variant3      CACAGTAAGCGATTATTTCAGAAACATCCGTGCGCATTCCATCCACCCCCGGGTGTCGGT      239
                   ************************************************************

opaJ               CGGCTACGACTTTGGCGGCTGGAGAATAGCGGCAGATTATGCCCGTTACAGAAAATGGAA      293
opaA_Variant1      CGGCTACGATTTCGGCGGCTGGAGGATAGCGGCAGATTATGCCAGTTACAGAAAATGGAA      299
opaB               CGGCTACGATTTCGGCGACTGGAGAATAGCGGCAGATTATGCCAGTTACAGAAAATGGAA      294
opaD               CGGCTACGATTTCGGCGACTGGAGAATAGCGGCAGATTATGCCAGTTACAGAAAATGGAA      294
opaA_Variant2      CGGCTACGATTTCGGCGGCTGGAGAATAGCGGCAGATTATGCCAGTTACAGAAAATGGAA      299
opaA_Variant3      CGGCTACGATTTCGGCGGCTGGAGGATAGCGGCAGATTATGCCAGTTACAGAAAATGGAA      299
                   ********* ** **** ****** ****************** ****************

opaJ               CAACAATAAATATTCCGTCAACACAAAAAATGTGCAAAAAAACGACAATGGCAACAGGCA      353
opaA_Variant1      CAACAATAAATATTCCGTCAACACAAAAGAGGTGCAAAGACATAGCAATGGCAACAGGGA      359
opaB               CAACAATAAATATTCCGTCAACACAAAAGAGTTGGAAAACAAG---CTTAACAATAAGAA      351
opaD               CAACAATAAATATTCCGTCAACACAAAAGAGTTGGAAAACAAG---CTTAACAATAAGAA      351
opaA_Variant2      CAACAATAAATATTCCGTCAACACAAAAGAGTTGGAAAACAAG---CTTAACAATAAGAA      356
opaA_Variant3      CAACAATAAATATTCCGTCAACACAAAAGAGGTGCAAAGACATAGCAATGGCAACAGGGA      359
                   **************************** *  ** ***   *      *  *** * * *

opaJ               AGACCTGAAGACGGAAAATCAGGAAAACGGTACATTCCACGCCGTTTCTTCTCTCGGCTT      413
opaA_Variant1      AGACCTGAAGACGGAAAATCAGGAAAACGGTACGTTCCACGCCGTCTCTTCTCTCGGCTT      419
opaB               AGACCTGAAGACGGAAAATCAGGAAAACGGTACATTCCACGCCGCTTCTTCTCTCGGCTT      411
opaD               AGACCTGAAGACGGAAAATCAGGAAAACGGTACATTCCACGCCGCTTCTTCTCTCGGCTT      411
opaA_Variant2      AGACCTGAAGACGGAAAATCAGGAAAACGGTACATTCCACGCCGCTTCTTCTCTCGGCTT      416
opaA_Variant3      AGACCTGAAGACGGAAAATCAGGAAAACGGTACATTCCACGCCGCTTCTTCTCTCGGCTT      419
                   ********************************* **********  **************

opaJ               ATCCGCCATTTACGATTTCAAACTCAACGATAAATTCGATAAATTCAAACCCTATATCGG      473
opaA_Variant1      ATCAGCCATTTACGATTTCAAACTCAACGATAAATTCGATAAATTCAAACCCTATATCGG      479
opaB               ATCCGCCATTTACGATTTCAAACTCAACGATAA---------ATTCAAACCCTATATCGG      462
opaD               ATCCGCCATTTACGATTTCAAACTCAACGATAA---------ATTCAAACCCTATATCGG      462
opaA_Variant2      ATCCGCCATTTACGATTTCAAACTCAACGATAA---------ATTCAAACCCTATATCGG      467
opaA_Variant3      ATCCGCCATTTACGATTTCAAACTCAACGATAA---------ATTCAAACCCTATATCGG      470
                   *** *****************************         ******************
```

```
opaJ              TGTGCGCGTCGCCTACGGACACGTCAGACACAGCATCGATTCGACCAAAAAAACAACAAA      533
opaA_Variant1     TGCGCGCGTCGCCTACGGACACGTTAAACATCAGGTTCATTCGGTGGAAACCAAAACCAC      539
opaB              TGCGCGCGTCGCCTACGGACACGTTAAACATCAGGTTCATTCGGTGGAAAGCAAAACCAC      522
opaD              TGCGCGCGTCGCCTACGGACACGTTAAACATCAGGTTCATTCGGTGGAAAGCAAAACCAC      522
opaA_Variant2     TGCGCGCGTCGCCTACGGACACGTTAAACATCAGGTTCATTCGGTGGAAAGCAAAACCAC      527
opaA_Variant3     TGCGCGCGTCGCCTACGGACACGTTAAACATCAGGTTCATTCGGTGGAAAGCAAAACCAC      530
                  ** ********************* * ***     *  *****    ***  * *** * 

opaJ              TGTTGTTACCGTCGCCGGT------GCTGCTAACACAGCACCTACGATTTATTATGCACC      587
opaA_Variant1     GATTGTTACCTCTAAACCAACGGGAGGTGCTACAGTGGGAGGCACTATCCC---AAGTTC      596
opaB              GATTGTTACCACTGCACCAACGCAAGGTGCTCTAAAGGGAGGCACTATCCTACAAGGTCC      582
opaD              GATTGTTACCACTGCACCAACGCAAGGTGCTCTAAAGGGAGGCACTATCCTACAAGGTCC      582
opaA_Variant2     GATTGTTACCACTGCACCAACGCAAGGTGCTCTAAAGGGAGGCACTATCCTACAAGGTCC      587
opaA_Variant3     GATTGTTACCACTGCACCAACGCAAGGTGCTCTAAAGGGAGGCACTATCCTACAAGGTCC      590
                    ********               * ****      * *   ** **           *

opaJ              AGAGACGCAAAACGCCTATCACGAAAGCCACAGCATCCGCCGCTTGGGTCTTGGTGTCAT      647
opaA_Variant1     GGTCATCAAACCTGCCTATCACGAAAGAAACAGCATCAGCAGCGTGGGTCTTGGTGTCAT      656
opaB              GACCCCCAAACCTCCCTATCACGAAAGCAACAGCATCAGCAGCTTGGGTCTTGGTGTCAT      642
opaD              GACCCCCAAACCTCCCTATCACGAAAGCAACAGCATCAGCAGCTTGGGTCTTGGTGTCAT      642
opaA_Variant2     GACCCCCAAACCTCCCTATCACGAAAGCAACAGCATCAGCAGCGTGGGTCTTGGTGTCAT      647
opaA_Variant3     GACCCCCAAACCTCCCTATCACGAAAGCAACAGCATCAGCAGCTTGGGTCTTGGTGTCAT      650
                          **    *************  ******** ** ** ****************

opaJ              CGCCGGTGTCGGTTTCGACATCACGCCCAAGCTGACCCTGGACACCGGATACCGTTACCA      707
opaA_Variant1     CGCCGGTGTCGGTTTCGACATCACGCCCAAGCTGACTTTAGACACCGGATACCGCTACCA      716
opaB              CGCCGGTGTCGGTTTCGACATCACGCCCAAGCTGACTTTAGACACCGGATACCGCTACCA      702
opaD              CGCCGGTGTCGGTTTCGACATCACGCCCAAGCTGACTTTAGACACCGGATACCGCTACCA      702
opaA_Variant2     CGCCGGTGTCGGTTTCGACATCACGCCCAAGCTGACTTTAGACACCGgaTACCGCTACCA      707
opaA_Variant3     CGCCGGTGTCGGTTTCGACATCACGCCCAAGCTGACTTTAGACACCGGATACCGCTACCA      710
                  ************************************  * ************** *****

opaJ              CAACTGGGGACGCTTGGAAAACACCCGCTTCAAAACCCACGAAGTCTCATTGGGCATGCG      767
opaA_Variant1     CAACTGGGGACGCTTGGAAAACACCCGCTTCAAAACCCACGAAGTCTCATTGGGCATGCG      776
opaB              CAACTGGGGACGCTTGGAAAACACCCGCTTCAAAACCCACGAAGTCTCATTGGGCATGCG      762
opaD              CAACTGGGGACGCTTGGAAAACACCCGCTTCAAAACCCACGAAGTCTCATTGGGCATGCG      762
opaA_Variant2     CAACTGGgGACGCTTGGAAAACACCCGCTTCAAAACCCACGAAGTCTCATTGGGCATGCG      767
opaA_Variant3     CAACTGGGGACGCTTGGAAAACACCCGCTTCAAAACCCACGAAGTCTCATTGGGCATGCG      770
                  ************************************************************

opaJ              CTACCGCTTCTGA  780
opaA_Variant1     CTACCGCTTCTGA  789
opaB              CTACCGCTTCTGA  775
opaD              CTACCACTTCTGA  775
opaA_Variant2     CTACCACTTCTGA  780
opaA_Variant3     CTACCGCTTCTGA  783
                  ***** *******
```

Supplementary Figure 1. Multiple sequence alignment of the *opa* alleles for the MenW:cc11 carriage isolates. Alignments of the nucleotide sequences of the *opaA*, *opaB*, *opaD* and *opaJ* variants as observed in the B285, R001 and R191 strains. Sequence identity is indicated with *. Nucleotide sequences were generated by dideoxy sequencing of PCR products spanning each of the four *opa* loci. Alignments were generated using Clustal Omega (1.2.4).

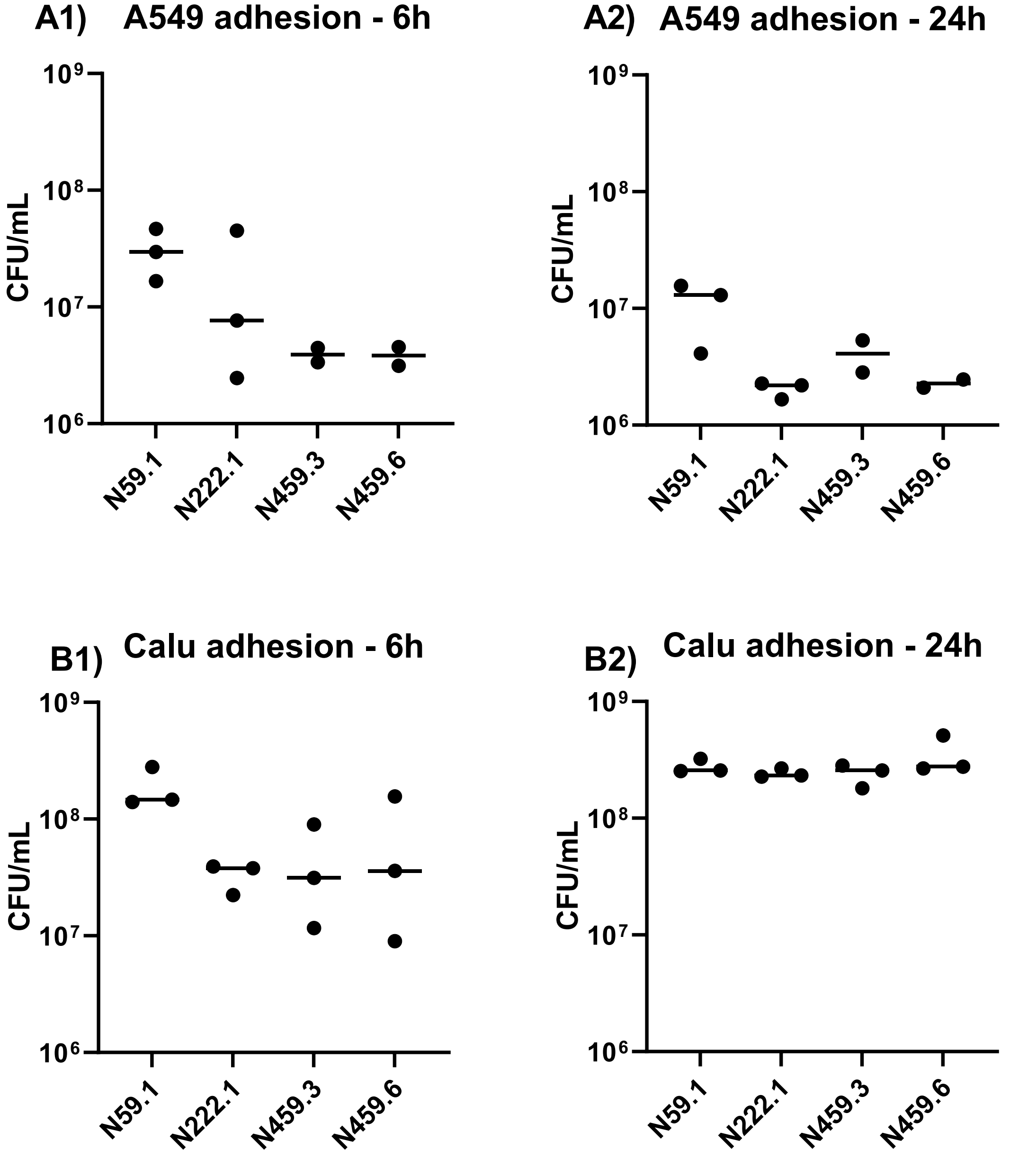

Supplementary Figure 2. Adherence of three MenY:cc23 carriage isolates from carrier V222 to A549 and Calu-3 cells. Semi-confluent A549 (A) or CaLu-3 (B) cells were infected with N222.1, N459.3, N459.6 and N59.1, at an MOI of 30 for 6 (A1, B1) or 24 (A2, B2) hours. After incubation, non-adherent bacteria were removed by sequential PBS washes and lysed with 0.1% saponin to release cell associated bacteria. Bacterial cfu was determined by plating serial dilutions on BHI agar plates. No significant differences were observed when each column was compared with each other (ordinary one way-ANOVA).

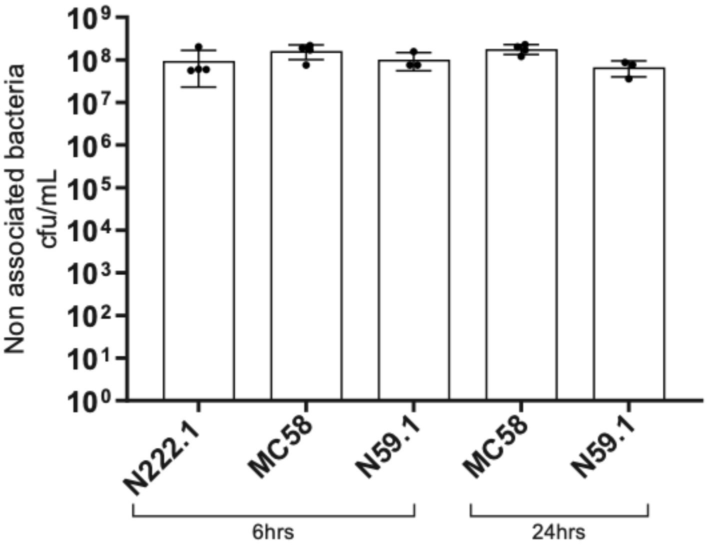

Supplementary Figure 3. Non-associated growth of N222.1, MC58 and N59.1 in the supernatants of ALI Calu3 monolayers. Calu-3 cells grown at ALI were infected with N222.1, MC58 or N59.1 at an MOI of 30 for 6 or 24 hours. The supernatant from the apical chamber was collected to calculate the number of non-associated bacteria present. Bacterial cfu counts were determined by plating serial dilutions on BHI agar plates. No data was obtained for the 24 hour time point with N222.1 due to complete disruption of the monolayer.

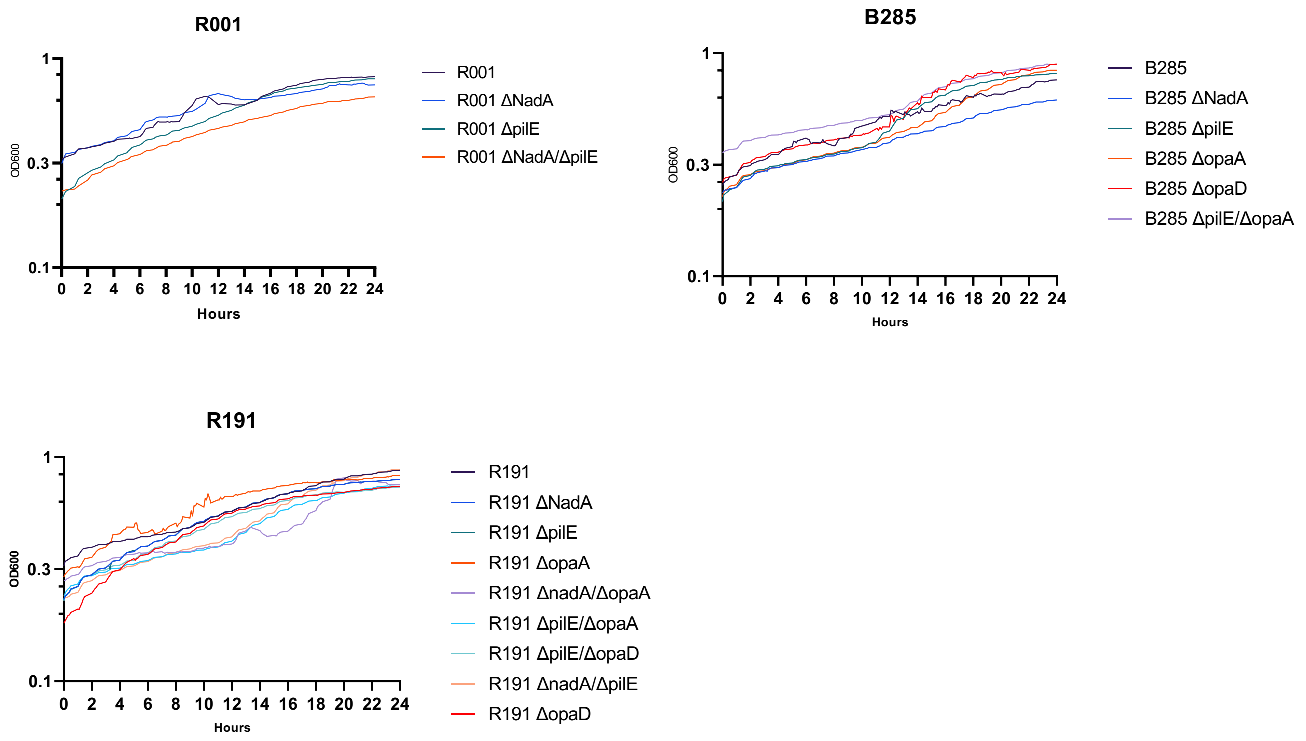

Supplementary Figure 4. Growth curves of MenW:cc11 R001, B285, and R191 wildtype and mutant strains in DMEM-F12 with 10% fetal bovine serum media. For growth curve multi-assay stock plates were prepared from exponentially growing isolates using our newly developed method (Farzand et al 2023). Briefly, the stock plate was defrosted and diluted (1:10) in DMEM-F12 with 10% FBS for 18 hr growth, which was then subcultured (1:10) into fresh DMEM-F12 with 10% FBS for actual growth measurement. The plate was incubated in Omega FLUOstar plate reader for 24 hrs and readings were taken every 10 minutes. A standard deviation of the mean was calculated for each time point from at least two independent biological experiment with 3 technical replicates each. Error bars were not included on the growth curves for clarity of presentation.

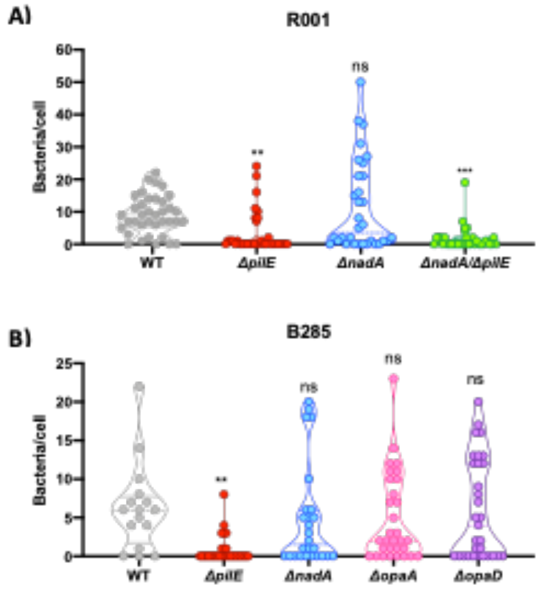

Supplementary Figure 5. Quantification of microcolony size by confocal microscopy analysis for meningococcal cells adhere to A549 cells. Microcolony formation on A549 cells was analysed after 19 hours of infection for the R001 and B285 strains (A and B, respectively). Adherent meningococcal cells were detected utilising a capsule-specific antibody and visualised by confocal microscopy. The numbers of meningococcal cells per individual A549 cell were quantified using Fiji program. At least 40 A549 cells were counted for each experimental condition on two independent occasions. Each symbol represents a single cell visible in the field of view. Statistical significance was tested for each mutant as compared to their respective WT strain. Significant differences are indicated: *, $P < 0.05$; **, $P < 0.01$ and ***, $P < 0.001$ (one-way ANOVA).

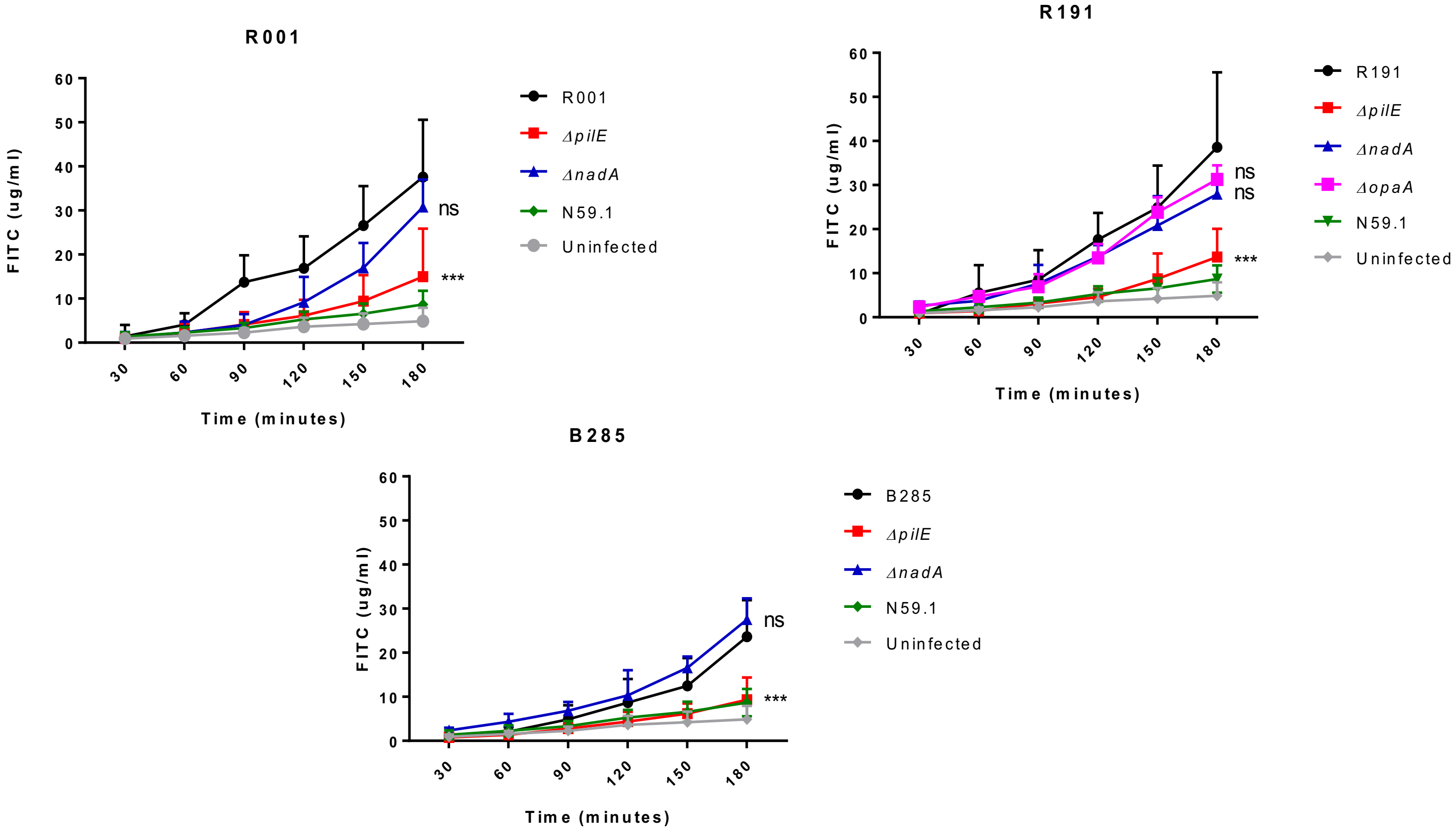

Supplementary Figure 6. Time course analysis of FITC-dextran permeation across Calu-3 monolayers. Calu-3 monolayers grown on transwell inserts were infected with MenW:cc11 (R001, R191 and B285) strains. FITC-dextran (1µg/ml) was added to the apical chamber and 100µl was taken from the basolateral chamber every 30 minutes. FITC-dextran was serially diluted from 250 µg to 0.4 µg in HBSS with 25 mM HEPES measured in the same plate as the test samples and used to generate a standard curve to convert raw fluorescence readings to concentrations of FITC-dextran. FITC-dextran was detected using an Omega FLUOstar plate reader with excitation and emission wavelengths of 485 nm and 520 nm, respectively. Error bars show the standard deviation of the mean from at least three independent biological replicates with technical duplicates. Significance values are reported for end-point (3 hours) fluorescence values compared to the corresponding WT strain. Strain N59.1 which is known to not disrupt monolayers was used as a negative control. Significant differences are indicated: *, $P < 0.05$; **, $P < 0.01$ ***, $P < 0.001$; and ****, $P < 0.0001$ (ordinary one way-ANOVA).

Supplementary Table 1. Oligonucleotide primer sequences.

Gene	Primer Name	Primer Sequences (5' – 3')	
		**Generation of Mutants**	
pilE	PilEMut_US_F<br>PilEMut_US_R	GCGTAGGGCTGATGTGTAGG<br>AGCTAGCTTGCATGGCCGTCTGAAGACAATGACCGGGTTTGACC	Upstream Flank
	PilEMut_DS_F<br>PilEMut_DS_R	ATTTAATAAGTAAGATGCCGTCTGAAAGCTCGATCAGGGTGAAACC<br>GCATTATTACGCCGCAAGCC	Downstream Flank
	PilEMut_Ery_F<br>PilEMut_Ery_R	GTCTTCAGACGGCCATGCAAGCTAGCTTTGGC<br>GCTTTCAGACGGCATCTTACTTATTAAATAATTTATAGCTATTG	Erythromycin Cassette
opa	OpaMut_US_F<br>OpaMut_US_R	GCAGGCGGCAAGTGAAGACG<br>CGCTGGGTTTATCGGCCGTCTGAAGGCATAATCTGCCGCTATCC	Upstream Flank
	OpaMut_DS_F<br>OpaMut_DS_R	AGATGTCTAAAAAGGCCGTCTGAAGCGCGTCGCCTACGGACACG<br>GCATGCCCAATGAGACTTCGTGG	Downstream Flank
	OpaMut_Kan_F<br>OpaMut_Kan_R	GCCTTCAGACGGCCGATAAACCCAGCGAACCATTTG<br>CGCTTCAGACGGCCTTTTTAGACATCTAAATCTAGGTAC	Kanamycin Cassette
		**Confirmation of *opa* allele mutant**	
opaA	OpaA_Flank_F<br>OpaA_Flank_R	GCAATAAACGAGCTGCTCCAG<br>GCATGCCCAATGAGACTTCGTGG	(Callaghan *et al*. 2006[1])
opaB	OpaB_Flank_F<br>OpaB_Flank_R	TTGAAACATCGCCCCAAACC<br>CGACCACTATTTCAGCACGG	
opaD	OpaD_Flank_F<br>OpaD_Flank_R	CCGCATTGATTTCGCGCGACA<br>GCATGCCCAATGAGACTTCGTGG	(Callaghan *et al*. 2006[1])
opaJ	OpaJ_Flank_F<br>OpaJ_Flank_R	TTGAAACATCGCCCCAAACC<br>CCGTAGCAGCAGACGACGTT	
		**PV analysis**	
opa	Opa_Con_F<br>Opa_Con_R	6FAM-GGATGGAATGCGCACGGATG<br>ATCCGCCTGCACATAATACGG	Amplifies all 4 opa alleles

[1]Callaghan, M. J., Jolley, K. A. and Maiden, M. C. J. (2006) 'Opacity-associated adhesin repertoire in hyperinvasive Neisseria meningitidis', *Infection and Immunity*. doi: 10.1128/IAI.00293-06.
